# Renal cell carcinoma risk among individuals heterozygous for fumarate hydratase variants: further insights into genotype-phenotype correlations

**DOI:** 10.1186/s13053-026-00342-1

**Published:** 2026-05-12

**Authors:** Trevor L. Hoffman, Sony Wirio, Vivek Sethumadhavan

**Affiliations:** 1 Department of Genetics, Southern California Kaiser Permanente Medical Group, Pasadena, CA USA; 2 Department of Pathology, Southern California Kaiser Permanente Medical Group, Pasadena, CA USA; 3 Bernard J. Tyson School of Medicine, Kaiser Permanente, Pasadena, CA USA

**Keywords:** FH-tumor predisposition syndrome, Hereditary leiomyomatosis and renal cell cancer, Fumarate hydratase, Renal cell carcinoma, Hereditary cancer, Cancer screening, Cancer prevention

## Abstract

**Background:**

FH–tumor predisposition syndrome (FH-TPS) is an autosomal dominant cancer predisposition syndrome caused by the fumarate hydratase (*FH*) gene. *FH* was initially shown to cause hereditary leiomyomatosis and renal cell cancer (HLRCC), historically called Reed syndrome. More recently, certain germline *FH* variants have been linked to pheochromocytoma (PHEO) risk in individuals without features of HLRCC. Diagnosis of these apparently non-overlapping conditions, FH-TPS-PHEO and FH-TPS-HLRCC, has increased through application of broad hereditary cancer gene panels. Early HLRCC studies suggested high lifetime RCC risk; however, emerging data suggests RCC risk in FH-TPS-HLRCC is lower than previous estimates.

**Methods:**

Individuals with likely pathogenic or pathogenic (LPV/PV) *FH* variants were identified in a large, ethnically diverse health system. Heterozygotes were classified as FH-TPS-PHEO, FH-TPS-HLRCC, or FH-ARC (autosomal recessive carrier for congenital fumarate hydratase deficiency without FH-TPS risk) based on variant phenotype-genotype prediction. Groups were evaluated for demographics, *FH* testing indications, FH-TPS-HLRCC related clinical features, and RCC. Cumulative RCC incidence was estimated via Aalen-Johansen estimator.

**Results:**

Among 279 individuals with LPV/PV in *FH*, 4 had FH-TPS-PHEO, 111 FH-TPS-HLRCC, and 164 FH-ARC. The FH-TPS-HLRCC and FH-ARC groups were approximately two-thirds female. FH-ARC was less ethnically diverse due to a common variant in Northern Europeans (c.1431_1433dup). One-third of FH-TPS-HLRCC individuals underwent testing for cancer-related indications unrelated to FH-TPS-HLRCC, and nearly 75% of these were women who had uterine leiomyomata suggestive of FH-TPS-HLRCC. One-quarter of FH-TPS-HLRCC individuals were diagnosed via cascade testing, 40% of whom had clinical features of FH-TPS-HLRCC. RCC occurred in nine patients (5 FH-TPS-HLRCC, 3 FH-ARC, 1 FH-TPS-PHEO). All RCCs in FH-TPS-HLRCC were FH-deficient and occurred at earlier average age than the sporadic RCC in other groups. Cumulative RCC incidence by age 70 was 7.3% (95% CI 2.1–15.3%) in FH-TPS-HLRCC and 2.6% (95% CI 0.0-7.1%) in FH-ARC. RCC risk before age 25 in FH-TPS-HLRCC was negligible.

**Conclusions:**

FH-TPS-HLRCC is underdiagnosed and RCC risk is lower than earlier HLRCC estimates. Many individuals are identified through broad hereditary cancer gene panel testing and exhibit less obvious manifestations such as uterine leiomyomata. Adult RCC surveillance is warranted in FH-TPS-HLRCC, but the need for pediatric screening merits further study.

**Supplementary Information:**

The online version contains supplementary material available at 10.1186/s13053-026-00342-1.

## Introduction

Fumarate hydratase (FH) is a key metabolic enzyme that catalyzes the reversible hydration of fumarate to L-malate within the tricarboxylic acid cycle. FH is localized both in the mitochondrial matrix and the cytosol, a dual distribution achieved through differential inclusion of an N-terminal mitochondrial targeting sequence as determined by alternate transcriptional initiation [1, 2]. In mitochondria, FH is essential for cellular ATP production and oxidative phosphorylation. Biallelic inactivating variants in *FH* lead to autosomal recessive (AR) fumarate hydratase deficiency—a rare metabolic disorder characterized by severe neurological impairment, primarily due to disrupted energy metabolism [3].

In contrast, cytosolic FH plays a distinct role in DNA damage repair, protection from double-strand DNA breaks, and acts as a tumor suppressor. Inactivating cytosolic variants in FH can predispose individuals to FH-tumor predisposition syndrome (FH-TPS), an autosomal dominant hereditary tumor predisposition syndrome comprised of two apparently allelic disorders with non-overlapping risk profiles: (1) individuals with increased risk for cutaneous/uterine leiomyomata and aggressive renal cell carcinoma (RCC), and (2) individuals with increased risk for pheochromocytoma [4]. In 2002, *FH* was found to be the genetic cause for the clinical entity hereditary leiomyomatosis and renal cell carcinoma (HLRCC), originally described in the literature as Reed syndrome [5, 6]. In 2014, several groups linked specific germline inactivating variants in *FH* to patients with pheochromocytoma who did not have features of HLRCC [7, 8]. In FH-TPS, tumorigenesis typically results from a somatic “second-hit” inactivation of a single functional *FH* allele, leading to fumarate accumulation, pseudohypoxia, and DNA damage that drive oncogenic transformation [9, 10]. In FH-deficient RCC, biallelic *FH* inactivation results in clear histological, immunostaining, and molecular characteristics [11].

Genotype-phenotype correlations have emerged for the various FH-related disorders. Some *FH* variants confer risk for AR *FH* deficiency without causing FH-TPS. A number of these AR specific variants disrupt the N-terminal mitochondrial targeting sequence while preserving cytosolic FH function through use of a downstream initiator methionine in the *FH* transcript, thereby maintaining tumor suppressor activity [1, 2]. These variants which alter the mitochondrial targeting sequence but preserve cytosolic enzyme function have low allele frequencies in the general population. Several other variants located C-terminal to the mitochondrial targeting sequence also selectively cause AR FH deficiency, including a common single amino acid insertion in the fumarase domain (c.1431_1433dup, p.Lys477dup), which is present in ~1/217 non-Finnish Europeans [12–14]. The mechanism by which this common single amino acid insertion and other less common missense variants cause AR disease but not FH-TPS is unknown, but may relate to impairments of cytosolic FH activity that do not reach a critical threshold to promote tumor formation [15]. By contrast, inactivating variants severely reducing or eliminating FH enzyme activity in all cellular compartments have been linked to FH-TPS in heterozygotes. Deletions, truncations, and nonsense variants resulting in haploinsufficiency typically result in an HLRCC-like presentation (FH-TPS-HLRCC) along with a number of inactivating missense variants [4]. Finally, a small number of unique missense inactivating *FH* variants have been linked to pheochromocytoma and paraganglioma risk without causing features of HLRCC (FH-TPS-PHEO) [4]. The specific mechanism(s) by which specific variants cause FH-TPS-HLRCC versus FH-TPS-PHEO is still not clear.

Identifying individuals heterozygous for LPV/PV *FH* variants and understanding disease-specific risks is critical for reproductive and hereditary cancer risk assessment and counseling. FH-TPS-HLRCC and FH-TPS-PHEO exhibit incomplete penetrance and variable expressivity, with FH-deficient RCC and FH-deficient pheochromocytoma representing potentially life-threatening manifestations of these allelic conditions, respectively [4]. While early studies suggested a lifetime RCC risk exceeding 30% in selected families with clinically-ascertained HLRCC (reviewed by [16]), more recent data driven by increased recognition and broader application of *FH* testing indicate that FH-TPS may be more prevalent and result in lower RCC risk than previously recognized [12, 17]. In this study, we aim to assess the lifetime RCC risk in a cohort of patients within a large health plan, comparing individuals with *FH* variants selectively associated with AR fumarate deficiency (FH-ARC) to those with variants linked to FH-TPS-PHEO and FH-TPS-HLRCC.

## Methods

Patients were identified who were heterozygous for germline LPV/PV variants in *FH* from a comprehensive database of approximately 57,000 hereditary cancer gene panels in a large, ethnically diverse health system with ~4.7 million patients in Southern California between 2014 and 2025 [18, 19]. Most individuals with LPV/PV in *FH* (> 98%) underwent a broad multigene hereditary cancer gene panel with 28 to 48 genes (including *FH*) designed to diagnose common hereditary breast, ovarian, pancreatic, colon, prostate, and/or renal cancer conditions per standard NCCN-based testing guidelines. In our organization, hereditary cancer testing is ordered by both genetics and non-genetics providers, about half of which is currently ordered by oncology-related non-genetics providers.

Patients with LPV/PV in *FH* were assigned to one of three groups based on the current understanding of the *FH* phenotype-genotype correlation: (1) *FH-ARC* (*A*utosomal *R*ecessive *C*arrier) group: heterozygous for LPV/PV *FH* variants which confer selective AR FH deficiency risk without conferring FH-TPS risk, (2) *FH-TPS-PHEO* group: heterozygous for LPV/PV in *FH* known to confer specific risk for FH-TPS-related pheochromocytoma, and (3) *FH-TPS-HLRCC* group: heterozygous for LPV/PV in *FH* known to confer risk for FH-TPS-related cutaneous/uterine leiomyomata and FH-deficient RCC. The strategy for variant group assignment is shown in Supplemental Fig. 1, and ClinVar identifications and variant information are presented in Supplemental Table 1. The FH-ARC group included: (a) all patients heterozygous for the common population variant c.1431_1433dup (p.Lys477dup), (b) all LPV/PV variants predicted to disrupt the N-terminal mitochondrial targeting sequence but retain cytosolic FH enzyme activity without ClinVar or literature association with FH-TPS, and (c) specific LPV/PV missense variants in *FH* with literature supporting selective AR association [3, 4]. The FH-TPS-PHEO group included all LPV/PV missense variants in *FH* associated in the literature with selective pheochromocytoma risk [4]. The FH-TPS-HLRCC group included all LPV/PV in *FH* predicted or known to cause FH-TPS based on prior association in the literature and/or ClinVar with HLRCC-related clinical features, and/or variants predicted to result in complete elimination of cytosolic and mitochondrial FH enzymatic activity (e.g. loss-of-function variants due to truncations, frameshifts, splice variants). Following variant assignment into the three groups by ClinVar and literature review, manual clinical chart review was performed to ensure: (1) patients with LPV/PV assigned to FH-ARC had no clinical features specific for FH-TPS-PHEO or FH-TPS-HLRCC, and (2) patients assigned to FH-TPS-PHEO had no clinical features to suggest FH-TPS-HLRCC. Finally, manual chart review of patients assigned to FH-TPS-HLRCC was performed to assess for presence of clinical features of HLRCC (cutaneous leiomyomata, FH-deficient RCC, and/or early-onset/numerous/large uterine leiomyomata) and ensure no patients had history of pheochromocytoma/paraganglioma. Clinical chart review was 100% concordant with the variant assignment process and no patients had clinical features to suggest incorrect group assignment, with the understanding that penetrance for FH-TPS is not 100% and many of the clinical features are age-dependent. The specific LPV/PV *FH* variants present in each group are shown in Supplemental Fig. 1. All germline testing for *FH* was done through a commercial laboratory using next-generation sequencing technology (GeneDx or Invitae labs, USA).

Patient demographic characteristics (age, biological sex, race/ethnicity) were determined by review of electronic medical records. Patient ethnicities were self-reported. All but two patients listed a single race/ethnicity designation, and the two patients who listed two race/ethnicity designations (both indicated white and Hispanic) were assigned to the Hispanic category. Patients with RCC from all groups were initially determined via an ICD-10 diagnosis code and text-based EMR query for current or past history of RCC (see supplemental methods for coding specifics). This query identified 9 total patients with RCC, which was verified by manual chart review (9/9 cases, 100%). The accuracy of the query to exclude RCC from patients was verified manually by chart review in 40 randomly selected patient charts in the FH-ARC cohort to verify negative predictive value of the search methodology and was verified in 40/40 (100%) cases. All patients in FH-TPS-PHEO and FH-TPS-HLRCC underwent clinical chart review to verify presence/absence of RCC. For deceased patients, manual chart review was performed to determine cause of death which was conclusively determined to be secondary to RCC or non-RCC associated cause in all cases (23/23 patients, 100%). Chart reviews also done to determine: (1) indication for *FH* germline testing at the time of test ordering, (2) completion rates of genetic counseling for *FH* LPV/PV, (3) review of 3-generation pedigree taken by genetics provider to determine clinical or molecular features consistent with FH-TPS in family members, (4) presence/absence of cutaneous/uterine leiomyomata, pheochromocytoma, and paraganglioma.

For patients with RCC, hematoxylin and eosin stains and FH immunohistochemistry (IHC) were performed on all tumor samples. Several samples also underwent IHC staining for s-2-succinocysteiene (2SC), which has 100% sensitivity for FH-deficient RCC [20, 21]. All RCC cases were reviewed by a board-certified anatomical pathologist (author SW), and each RCC was designated FH-deficient RCC (FH-TPS-HLRCC associated) or non-FH-deficient RCC (sporadic) based on 2022 WHO RCC tumor classification criteria [11].

Statistical analysis for demographic comparisons between groups was performed using standard methodologies for Welch’s t-test, Chi-square, and Fisher’s exact test. Competing risk analyses were conducted using the scikit-survival package in Python (version 0.24.1). The cumulative incidence function (CIF) was used to estimate the probability of RCC to account for death as a competing event that precedes the occurrence of RCC. The analysis treated three mutually exclusive outcomes: RCC diagnosis, death without prior RCC diagnosis, and censored observations (individuals alive and without RCC at the end of follow-up). CIF curves were plotted as step functions across observed ages for the FH-ARC and FH-TPS-HLRCC groups using the Aalen-Johansen estimator with log-log confidence intervals, which extends the Kaplan-Meier method to the competing risks setting. The CIF represents the probability of experiencing renal cell carcinoma by a given age, accounting for the competing risk of death. Specifically, the CIF at time t is defined as: CIF(t) = ∫₀ᵗ S(u⁻) dΛ₁(u). S(u) is the overall survival probability (free from all events) and Λ₁(u) is the cause-specific cumulative hazard for renal cell carcinoma. The cumulative incidence of RCC was extracted from the CIF using linear interpolation between estimated time points. To account for sampling variability, 95% confidence intervals were constructed using non-parametric bootstrap with 1,000 resamples. For each bootstrap iteration: (1) random samples were drawn within the FH-ARC and FH-TPS-HLRCC groups using the observed group sample size, (2) CIF was computed at age 70 for resampled data, and (3) valid estimates were retained (excluding numerical failures). The 95% confidence interval was defined by the 2.5th and 97.5th percentiles of the bootstrap distribution. This non-parametric approach makes no distributional assumptions and is appropriate for small sample sizes where asymptotic methods may be unstable. Bootstrap resampling provides a more stable empirical estimate of uncertainty in datasets with sparse events.

## Results

### Identification of FH-ARC and FH-TPS-PHEO, and FH-TPS-HLRCC variant groups

A database search for all patients with LPV/PV in *FH* who underwent hereditary cancer gene panel testing in a large health system from a period of just over 10 years and representing approximately 57,000 test results (2014–2025) revealed 279 patients who were heterozygous for a LPV/PV in *FH*. No patients were identified who had AR FH deficiency due to biallelic *FH* LPV/PV. Among heterozygous individuals, 164 were heterozygous for a LPV/PV in *FH* predicted to cause AR disease risk without causing FH-TPS (FH-ARC group). A total of 9 distinct AR variants were identified, including the well-described c.1431_1433dup (p.Lys477dup) variant which is common in the general population (*n* = 146/164 in FH-ARC group), 4 LPV/PV affecting the N-terminal mitochondrial targeting sequence of *FH* that would be predicted to result in a functional cytosolic enzyme (*n* = 9/164 in FH-ARC group), and 4 missense LPV/PV specifically associated with AR disease (*n* = 9/164 in FH-ARC group). A total of 4 individuals were heterozygous for LPV/PV predicted to cause FH-TPS-PHEO from 3 independent *FH* variants. A total of 111 individuals were heterozygous for *FH* LPV/PV predicted to cause FH-TPS-HLRCC, comprised of 25 variants predicted to result in haploinsufficiency (splice-site, exon/multi-exon deletions, nonsense, frameshift variants; *n* = 44/111 in FH-TPS-HLRCC group), one in-frame 7 amino acid deletion associated with FH-TPS-HLRCC (c.786_806del, p.Lys263_Ile269del; *n* = 1/111 in FH-TPS-HLRCC group), and 32 missense variants known to cause clinical features of FH-TPS-HLRCC in the literature (*n* = 66/111 in FH-TPS-HLRCC group). All specific variants observed and assigned to the three groups are shown in Supplemental Fig. 1.

### Additional hereditary cancer genetic conditions among FH groups

Among patients in FH-ARC, FH-TPS-PHEO, and FH-TPS-HLRCC groups, a total of 21 patients were found to have an LPV/PV in another hereditary cancer gene unrelated to *FH* (21/279 = 7.5% of total patient cohort). A total of 14 patients in the FH-ARC group (14/164 = 8.5% of FH-ARC group) had a diagnosis of hereditary cancer unrelated to *FH* (*n* = 1 *ATM*, *n* = 4 *BRCA1*, *n* = 2 *BRCA2*, *n* = 1 *CDKN2A*, *n* = 2 *CHEK2*, *n* = 1 *MITF*, *n* = 1 *NF1*, *n* = 1 *RAD51C*, and *n* = 1 with *BRCA1/CHEK2*; all LPV/PV). One patient in the FH-TPS-PHEO group (1/4 = 25% of FH-TPS-PHEO group) had a diagnosis of hereditary cancer risk unrelated to FH (*n* = 1 APC). A total of 6 patients in the FH-TPS-HLRCC group (6/111 = 5.4% of FH-TPS-HLRCC group) had an additional hereditary cancer diagnosis unrelated to *FH* (*n* = 1 *APC* risk allele, *n* = 1 *BRCA1*, *n* = 1 *BRCA2*, *n* = 2 *CHEK2*, *n* = 1 *PMS2*).

### Demographics of FH-ARC and FH-TPS variant groups

An analysis of the demographic characteristics of the FH-ARC, FH-TPS-PHEO, and FH-TPS-HLRCC groups was performed and is presented in Table 1. Due to the small number of patients in the FH-TPS-PHEO group (*n* = 4), further demographic subgroup and statistical comparison were limited to FH-ARC and FH-TPS-HLRCC groups, though FH-TPS-PHEO patients were included in the total *FH* variant analysis. The mean age for all patients was 56.2 years and was younger in the FH-TPS-HLRCC group (51.1 years) than the FH-ARC group (59.9 years; *p* < 0.01 Welch’s t-test). The fraction of females was similar in both groups, representing 67% of all patients (68% of FH-ARC vs. 66% of FH-TPS-HLRCC group; not significant). The distribution of ethnicities between groups revealed a higher fraction of FH-ARC patients reporting White ethnicity (84% FH-ARC vs. 46% FH-TPS-HLRCC), while the FH-TPS-HLRCC group had a higher percentage of Hispanic/Latino (27% FH-TPS-HLRCC vs. 8% FH-ARC), Black (13% FH-TPS-HLRCC vs. 4% FH-ARC), and Asian (7% FH-TPS-HLRCC vs. 0% FH-ARC) individuals. The differences in ethnicities between FH-TPS-HLRCC and FH-ARC groups reached statistical significance (Chi-square; *p* < 0.0001) and likely relates to the effect of the c.1431_1433dup (p.Lys477dup) as a common population variant in N/W European individuals [14].

**Table 1:** Demographic characteristics of patient groups

Characteristic	Total (*N* = 279)	FH-ARC (*n* = 164)	FH-TPS-HLRCC (*n* = 111)	FH-TPS-PHEO (*n* = 4)
**Mean Age** (SD)	56.2 (17.1)	59.9 (15.9)	51.1 (17.6)	44.8 (12.1)
**Age Subgroups** (n, % of group)				
* ≤* 17	2 (1%)	0 (0%)	2 (2%)	0 (0%)
18 to 29	15 (5%)	4 (2%)	11 (10%)	0 (0%)
30 to 39	36 (13%)	16 (10%)	18 (16%)	2 (50%)
40 to 49	54 (19%)	31 (19%)	22 (20%)	1 (25%)
50 to 59	41 (15%)	22 (13%)	19 (17%)	0 (0%)
60 to 69	59 (21%)	39 (24%)	19 (17%)	1 (25%)
70 to 79	55 (20%)	39 (24%)	16 (14%)	0 (0%)
80 to 89	13 (5%)	10 (6%)	3 (3%)	0 (0%)
90 and older	4 (1%)	3 (2%)	1 (1%)	0 (0%)
**Biological Sex**				
Female	187 (67%)	112 (68%)	73 (66%)	2 (50%)
Male	92 (33%)	52 (32%)	38 (34%)	2 (50%)
**Race/Ethnicity**				
White	192 (69%)	137 (84%)	51 (46%)	4 (100%)
Hispanic/Latino	43 (15%)	13 (8%)	30 (27%)	0 (0%)
Black	20 (7%)	6 (4%)	14 (13%)	0 (0%)
Unknown	13 (5%)	7 (4%)	6 (5%)	0 (0%)
Asian	8 (3%)	0 (0%)	8 (7%)	0 (0%)
Native Hawaiian/Other Pacific Islander	1 (0%)	1 (1%)	0 (0%)	0 (0%)
Other	2 (1%)	0 (0%)	2 (2%)	0 (0%)

### Genetic counseling, indications for *FH* testing, and relevant clinical findings in FH-TPS group

The completion of genetic counseling for all patients with LPV/PV in *FH* was examined. A total of 271/279 (97%) patients received genetic counseling regarding their LPV/PV *FH* variant by a genetics specialist (medical genetics physician or genetic counselor). Of the 8 patients that did not complete genetic counseling, 7/8 were in the FH-ARC group and 1/8 was in the FH-TPS-HLRCC group. All 7 patients who did not complete genetic counseling in the FH-ARC group lacked additional LPV/PV on their hereditary cancer gene panel, and all 7 had a primary cancer diagnosis unrelated to FH-TPS. The failure of the 7 patients in the FH-ARC group to complete genetic counseling could be due to the results being perceived as relevant only for reproductive counseling by the ordering provider and/or patient (and not relevant for their active cancer care), but this was not formally examined. The single patient who did not receive post-test genetic counseling from the FH-TPS-HLRCC group declined a referral to genetics for reasons that were also not clear from chart review.

Test indications, FH-TPS related clinical features, and relevant family history were further examined for FH-TPS-PHEO and FH-TPS-HLRCC groups. For the 4 patients in the FH-TPS-PHEO group, 2 had no primary testing indication related to FH-TPS, while 2 had an FH-TPS-related indication (1 with RCC, which was ultimately determined to be sporadic, Table 2; 1 for cascade testing due to a family member with FH-TPS-PHEO DNA test result). None of the 4 patients in the FH-TPS-PHEO group had a family history with clinical features of FH-TPS-PHEO.

**Table 2:** Analysis of RCC in patients with *FH* LPV/PV

Sex	Status	Age at Death or follow up	Death from RCC?	Age at RCC Dx	RCC Pathology	FH IHC	2SC IHC	Germline FH variant	Group	RCC Type	FH-TPS-HLRCC Derm	FH-TPS-HLRCC Uterine	FH-TPS-HLRCC FHx
M	D	54	Y	52	Clear cell	Int	Weak, patchy cytoplasmic	c.1431_1433dup (p.Lys477dup)	FH-ARC	Spor	N	N/A	N
M	D	70	Y	69	Poorly differentiated	Int	Neg	c.1431_1433dup (p.Lys477dup)	FH-ARC	Spor	N	N/A	N
M	A	71	N/A	72	Clear cell	Int	Neg	c.1431_1433dup (p.Lys477dup)	FH-ARC	Spor	N	N/A	N
M	A	44	N/A	37	Chromophobe	Int	Neg	c.222 A>T (p.Arg74Ser)	FH-TPS- PHEO	Spor	N	N/A	N
M	D	28	Y	28	Papillary	Def	ND	c.1108+1G>T	FH-TPS-HLRCC	FH def RCC	N	N/A	N
M	A	35	N/A	29	Papillary	Def	ND	c.1108+1G>T	FH-TPS-HLRCC	FH def RCC	N	N/A	N
F	A	39	N/A	37	Papillary	Def	Strongly positive in nucleus and cytoplasm	c.1268T>G (p.Leu423Arg)	FH-TPS-HLRCC	FH def RCC	N	Y	N
F	D	41	Y	39	Tubulocystic	Def	ND	c.1293delA (p.Glu432LysfsX17)	FH-TPS-HLRCC	FH def RCC	N	Y	N
M	A	67	N/A	62	Papillary, solid, cribriform/sieve-like	Int	ND	c.698G>A (p.Arg233His)	FH-TPS-HLRCC	FH def RCC	Y	N/A	Y

All RCC are presented. Demographic, patient group, renal pathology, and clinical features are listed for each patient. The absence of FH IHC is 100% specific for FH-deficient RCC, but ~10% FH-deficient RCC will demonstrate retained FH IHC [20, 21]. 2SC IHC has 100% sensitivity for FH-deficient RCC, and FH-deficient tumors demonstrate strongly positive 2SC staining in nucleus and cytoplasm, while FH-intact tumors will demonstrate negative or weak cytoplasmic 2SC staining [20, 21]. For the case represented on the last row, the combination of histological and molecular features met 2022 WHO criteria for FH-deficient RCC. This RCC also underwent tumor NGS showing very high (>80%) variant allele frequency for *FH* c.698G>A (p.Arg233His) consistent with whole-gene *FH* deletion of the opposite allele [11]. FH-TPS-HLRCC Derm column represents the presence of biopsy-confirmed cutaneous leiomyomata, while the FH-TPS-HLRCC Uterine column represents presence of early-onset, (+/- large) uterine leiomyomata. FH-TPS-HLRCC FHx column represents review of 3-generation pedigree taken by a medical geneticist or genetic counselor and the presence of any features suggestive of FH-TPS-HLRCC. Abbreviations: A (alive), D (deceased), Def (deficient), FHx (family history), Int (intact), N (no), N/A (not applicable), ND (not done), Neg (negative), Spor (sporadic), Y (yes)

**Fig. 1:**
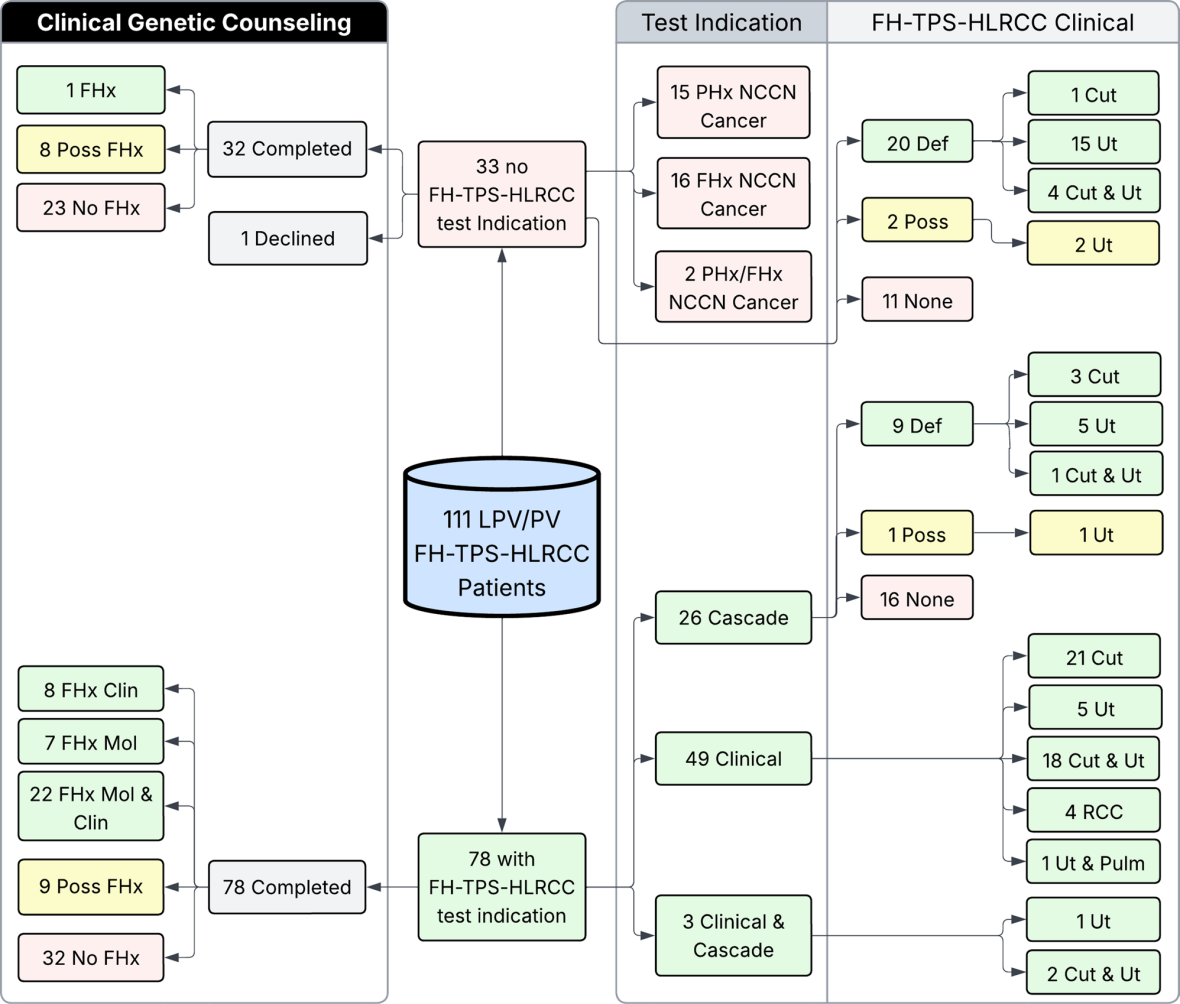
FH-TPS-HLRCC group test indications, clinical features, and family history. Swim lane diagram demonstrating family history information (left vertical lane, black heading), germline testing indications (middle vertical lane, dark grey heading), and clinical features indicative of HLRCC (right vertical lane, light grey heading) in the FH-TPS-HLRCC group. The green, yellow, and red colors within each lane represent present, possible, and absent elements, respectively, for each lane. For test indications, “NCCN Cancer” refers to NCCN-based personal- or family history-specific guidelines for germline testing for hereditary breast, ovarian, colon, pancreatic, and/or prostate cancer. Cascade indication represents a referral for germline testing based on a family member with a molecular diagnosis of FH-TPS-HLRCC. For clinical features, uterine findings suggestive of FH-TPS-HLRCC were considered present in female patients with history of uterine leiomyomata before age 40 years (including large lesions). If a patient reported undergoing hysterectomy for leiomyomata at an early age, this was considered a possible feature of FH-TPS-HLRCC when surgical pathology or imaging records were not available to quantify number/size. Cutaneous features represent biopsy-proven leiomyomata from skin (or in the case of one patient, pulmonary leiomyomata from lung biopsy). A clinical family history represents clinical features suggestive of FH-TPS-HLRCC while a molecular family history represents the patient providing records of a family member with DNA test results diagnostic of FH-TPS-HLRCC. Abbreviations: Clin (clinical), Cut (cutaneous leiomyomata), Def (definite), FHx (family history), Mol (molecular), NCCN (National Comprehensive Care Network), PHx (personal history), Poss (possible), Pulm (pulmonary), RCC (renal cell carcinoma), Ut (uterine leiomyomata)

Among patients in the FH-TPS-HLRCC group, 33 had no test indication related to FH-TPS-HLRCC (33/111 = 30% of FH-TPS-HLRCC group), while 78 had an FH-TPS-HLRCC-related indication (78/111 = 70%; Fig. 1). All 33 FH-TPS-HLRCC patients without a primary FH-TPS-HLRCC test indication underwent testing due to a personal and/or family history of breast, ovarian, pancreatic, colon and/or prostate cancer under NCCN guidelines (Fig. 1). Thus, the FH-TPS-HLRCC patients identified who were lacking an FH-TPS-related testing indication were not necessarily expected and might be considered “incidental findings”. Interestingly, 61% (20/33) of the FH-TPS-HLRCC patients lacking FH-TPS-related test indication had clinical manifestations suggestive of FH-TPS-HLRCC on chart review, mostly representing uterine leiomyomata (19/33 = 58%; 19/27 = 70% of females in this subgroup) but occasionally cutaneous features were present or were identified after molecular diagnosis (5/33 = 15%; Fig. 1). One-third (11/33 = 33%) of FH-TPS-HLRCC patients without FH-TPS related test indication lacked any clinical features of FH-TPS-HLRCC, and 70% lacked a family history suggestive of FH-TPS-HLRCC (23/33 = 70%; Fig. 1).

For FH-TPS-HLRCC patients with an FH-TPS relevant indication for testing, the indication was clinical in 66% (52/78) and cascade (reporting or providing DNA test results from a family member) in 37% (29/78; note 3 patients had both clinical and cascade indications; Fig. 1). The most common clinical manifestation among FH-TPS-HLRCC patients with relevant indication for testing was cutaneous leiomyomata (45/78 = 58%), followed by uterine leiomyomata (33/78 = 42%), and RCC (4/78 = 5%). For the 26 FH-TPS-HLRCC patients undergoing testing solely for cascade reasons, 61% (16/26) lacked clinical manifestations. A family history suggestive of FH-TPS-HLRCC was present in 47% (37/78) of patients undergoing testing for FH-TPS-HLRCC-related indication (Fig. 1).

### Determination of sporadic vs. FH-TPS-associated RCC

RCC occurred in a total of 9 patients in both groups (3 from FH-ARC, 5 from FH-TPS-HLRCC, and 1 from FH-TPS-PHEO) which are reviewed in Table 2. A total of 4 cases (3 from FH-ARC group, 1 from FH-TPS-PHEO group) were assigned as sporadic RCC due to a combination of histopathology (e.g. chromophobe, clear cell, poorly differentiated histology) and normal IHC staining patterns for FH and 2SC. The histology IHC staining patterns for FH and 2SC are able to exclude FH-deficient RCC in these 4 cases with essentially 100% accuracy [21]. A total of 5 cases met 2022 WHO criteria for FH-deficient RCC (FH-TPS-associated RCC), all of which occurred in the FH-TPS-HLRCC group [11]. All 5 FH-deficient RCC had papillary or tubulocystic pathology, and 4/5 demonstrated deficient FH IHC staining. One case demonstrated intact FH IHC but met 2022 WHO criteria for FH-deficient RCC due to the presence of tumor morphology (multi-nodular and cystic RCC), histology (multiple admixed morphological patterns including papillary, solid, and cribriform/sieve-like along with prominent enlarged vesicular nuclei and eosinophilic macro-nucleoli) and molecular features (non-functional *FH* germline/tumor variant p.Arg233His with high variant allele frequency shown previously to result in retained FH IHC) [11, 20]. It has been previously shown that ~10% of FH-deficient RCC will have retained FH IHC [20, 21].

A statistical analysis of the types of RCC revealed that the number of sporadic (3 in FH-ARC vs. 0 in FH-TPS-HLRCC) and total RCC (3 in FH-ARC vs. 5 in FH-TPS-HLRCC) did not reach statistical significance between groups (*p* = 0.28 and 0.28, respectively), while the difference in FH-deficient RCC did reach statistical significance (0 in FH-ARC vs. 5 in FH-TPS-HLRCC; *p* = 0.01; two-tailed Fisher’s exact test). The average age at RCC diagnosis occurred earlier in the FH-TPS-HLRCC group (39.0 *±* 13.7 years) than in the FH-ARC group (64.3 *±* 10.8 years) which also reached statistical significance (*p* = 0.03, Welch’s t-test).

### Causes of death

Among both groups, 256 individuals were alive during the study period, while 23 were deceased. For the 23 deceased individuals, a total of 6 were from the FH-TPS-HLRCC group, 0 were from the FH-TPS-PHEO group, and 17 were from the FH-ARC group. Manual chart review was completed for all deceased patients to determine a specific cause of death. A total of 4/23 patients died from RCC (2 from FH-ARC group, 2 from FH-TPS-HLRCC group), while 19/23 died from a non-RCC associated cause (15/23 died from metastatic non-RCC cancer such as pancreatic, prostate, breast, gastrointestinal; 4/23 died secondary to causes unrelated to cancer).

### Lifetime RCC risk analysis

**Fig. 2:**
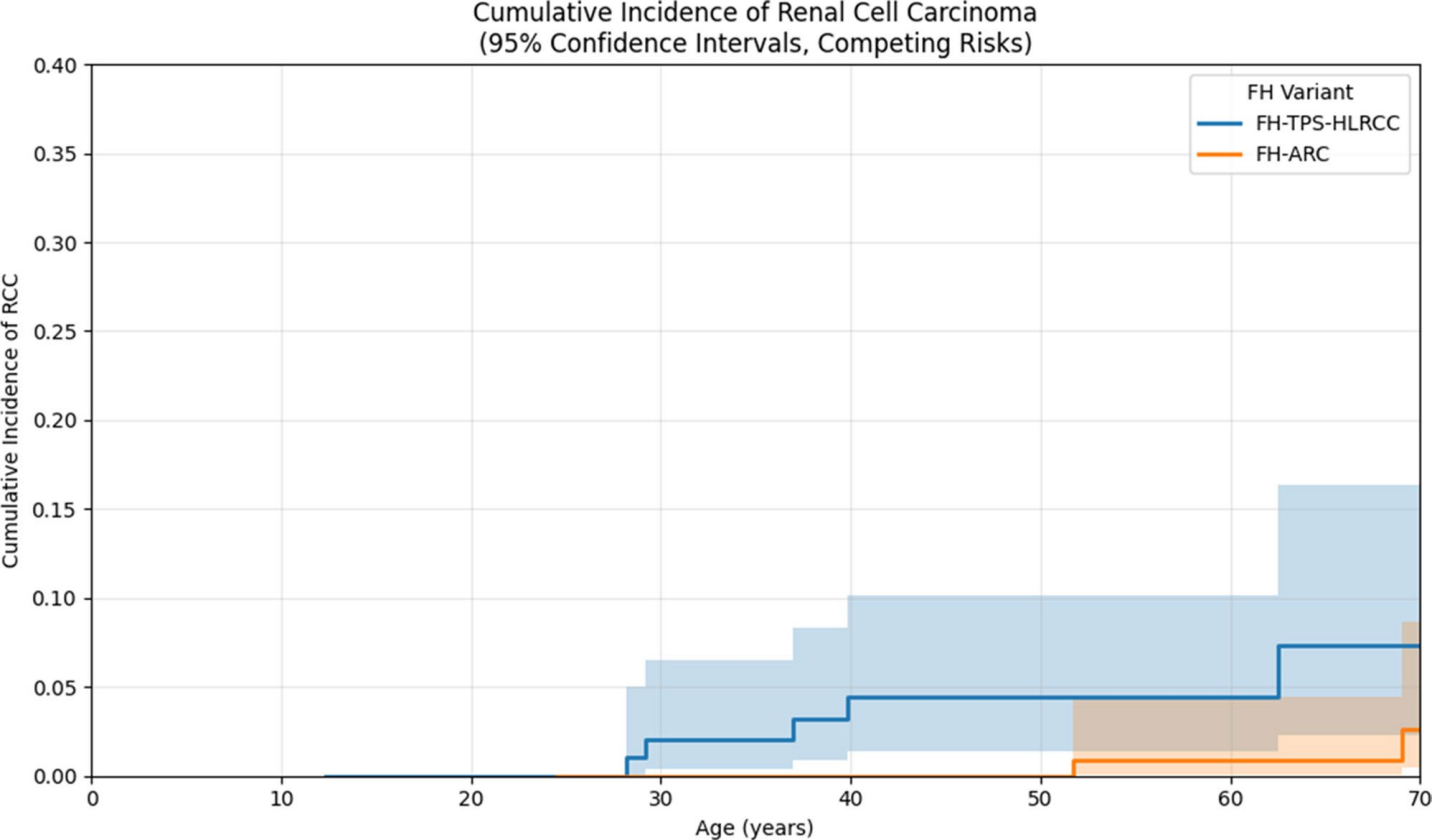
Cumulative incidence of renal cell carcinoma. Cumulative incidence curves with competing risks are shown for RCC in FH-TPS-HLRCC (blue line) and FH-ARC (orange line) groups as a function of patient age. Due to the low number of FH-TPS-PHEO patients, CIFs for this group could not be calculated with accuracy. The shading for each curve represents the 95% confidence intervals for CIF estimates

Using clinical information from the electronic medical record, we computed RCC risk in FH-ARC and FH-TPS-HLRCC groups using the Aalen-Johansen CIF estimator, which extends the Kaplan-Meier method to a competing risks framework. The CIF curves for RCC in both groups are shown in Fig. 2. Due to the small number of patients in the FH-TPS-PHEO group (*n* = 4), CIF calculations were not performed in this group. The cumulative incidence of RCC at age 70 years was 2.6% in the FH-ARC group (95% confidence interval 0.0% − 7.1%) and 7.3% in the FH-TPS-HLRCC group (95% confidence interval 2.1% − 15.3%). CIF calculations were not calculated beyond age 70 years due to the small number of patients over this age in both groups.

## Discussion

Lifetime risk estimates for hereditary cancer syndromes typically evolve through predictable stages. Early data, often derived from specialty clinics and severely affected families, tend to overestimate risk due to ascertainment bias. Before the availability of gene-specific testing, syndromes are frequently defined solely through clinical features, leading to preferential identification of highly penetrant and/or severe cases. As molecular diagnosis becomes possible and syndrome awareness expands, more representative and heterogeneous cohorts emerge, revealing a broader spectrum of penetrance prompting downward revisions of risk estimates. This trend has been well documented across numerous hereditary cancer syndromes, including *CDH1*-associated hereditary diffuse gastric cancer [22], hereditary paraganglioma conditions (especially *SDHA*) [23], *BRCA1/2*-associated hereditary breast and ovarian cancer [24], and Lynch syndrome (*MLH1*, *MSH2/6*, *PMS2*) [25]. Each of these conditions has undergone substantial refinement of risk estimates through this process.

FH-deficient RCC risk estimates for FH-TPS-HLRCC appear to be following the same trajectory. Available data remain limited, with only about ~1200–1500 affected individuals reported in the literature, and risk estimates are disproportionately influenced by premolecular data or restricted testing of clearly symptomatic patients. These early studies reported lifetime RCC risks ~15–35%, often without applying rigorous epidemiological methodology [16, 26–28]. However, more recent studies that incorporate molecular-based strategies to diagnose FH-TPS-HLRCC and apply modern statistical modeling consistently show lower estimates (~2–17%) [12, 17, 29]. Population genetic database analysis provides the lowest estimates to date (~2–12%) [17].

Our cohort, identified through broad germline hereditary cancer testing in a large integrated health system, includes both phenotype- and genotypic-driven diagnoses of FH-TPS-HLRCC. We estimate a cumulative RCC incidence of ~7.3% (95% CI 2.1–15.3%) at age 70 among patients with FH-TPS-HLRCC, aligning with recent estimates [12, 16, 17]. Our data also reinforce the lack of FH-deficient RCC risk in FH-ARC-specific variants of *FH*, including the common population c.1431_1433dup (p.Lys477dup) variant. The ~2.6% (95% CI 0.0-7.1%) cumulative RCC incidence in the FH-ARC group is comparable to general population RCC risk estimates of ~2% [30]. A key strength of our study is our detailed clinical data, which enables precise clinical and RCC phenotyping– information often unavailable in population datasets or commercial lab cohorts.

An emerging trend in the clinical setting is expanding use of broad multigene panels, which increase diagnostic yield and often uncover unexpected hereditary cancer diagnoses. Several studies demonstrate that broad multigene panels frequently identify LPV/PV unrelated to the patient’s personal or family cancer history (reviewed in [31]). Approximately 1–2% of individuals undergoing multigene hereditary cancer gene panel testing have multi-locus inherited neoplasia syndrome (MINAS), which is the presence of two or more germline pathogenic variants in different hereditary cancer predisposition genes, and ~5–10% of individuals with a single germline pathogenic variant harbor a second, independent one [32–35]. Our data are consistent with this finding, with 6% (7/115) of the combined FH-TPS patients in our study fulfilling MINAS criteria. *FH* has been underrepresented in many such studies, but as a “moderate risk” gene with incomplete penetrance and variable expressivity, diagnosis of FH-TPS is likely to continue increasing via this trend. In our cohort, approximately one-third of FH-TPS-HLRCC cases were diagnosed in patients undergoing testing without an FH-TPS-HLRCC-relevant indication, and patients diagnosed without an indication (or an unrecognized one) often lacked a suggestive family history and had fewer obvious clinical manifestations. Nearly 75% of the female patients diagnosed with FH-TPS-HLRCC who underwent hereditary cancer testing for reasons unrelated to FH-TPS-HLRCC in our study had definite or suggestive uterine findings that appeared to be unrecognized by ordering clinicians.

It is likely that FH-TPS-HLRCC is under-recognized and under-diagnosed for numerous reasons including: (1) many clinicians do not recognize uterine manifestations as suggestive of FH-TPS-HLRCC, (2) pathologists may overlook histological features of FH-deficient leiomyomata, (3) cutaneous leiomyomata are often asymptomatic and therefore may not be brought to medical attention, (4) many clinicians fail to biopsy cutaneous leiomyomata because they appear “benign” (author TH, personal observations), and/or (5) there is not universal IHC screening for FH-TPS-HLRCC in RCC or uterine leiomyomata [16, 17, 36]. Despite these challenges, FH-TPS-HLRCC clearly confers meaningful risk. In our cohort, 4/5 individuals with FH-deficient RCC were diagnosed with FH-TPS-HLRCC due to early-onset RCC, lacking cutaneous manifestations and family history. Because FH-deficient RCC is aggressive, timely surveillance and diagnosis remain critical.

The age at which RCC risk becomes clinically relevant is an important consideration for screening guidelines. Pediatric RCC in FH-TPS-HLRCC appears to be extremely rare. We found 11 cases of RCC in pediatric patients in published literature which could be verified in a peer-reviewed manuscript (Table 3). One patient had a congenital form of bilateral polycystic kidney disease which is not associated with FH-TPS-HLRCC and likely represents an independent diagnosis [37]. Other cases were not well-characterized (e.g., lacking description of RCC pathology and/or germline *FH* results), and virtually none have been reported to have undergone additional germline analysis for hereditary cancer conditions beyond *FH* (see Table 3 for references). Because ~5–10% of patients with a single hereditary cancer diagnosis likely have MINAS, it is quite possible that these patients with very-early onset RCC might have additional hereditary cancer conditions that create added risk. In our cohort, no FH-deficient RCC occurred before age 28 years and cumulative incidence of RCC was 0% under age 25. Although our sample size is modest, the consistency of published evidence suggests FH-deficient RCC is an ultrarare event, and it is possible that some very-early-onset RCC cases in the literature may reflect MINAS rather than FH-TPS-HLRCC alone.

**Table 3:** Pediatric RCC in HLRCC/FH-TPS-HLRCC in literature

Dx Age	RCC Pathology	Germline FH Variant	FH IHC	Additional Germline Analysis?	Reference
7	P/T	NM_000143.4(*FH*): c.378+1G>A	Deficient	WES	[38]
10	NS	NM_000143.4(*FH*): c.1118A>G (p.Asn373Ser)	ND	No	[29, 39]
11	P	NM_000143.4(*FH*): c.1189G>A (p.Gly397Arg)	ND	No	[40]
13, 16	P	ND	ND	No	[41]
15	P/T	NM_000143.4(*FH*):c.1301G>A (p.Cys434Tyr)	Deficient	No	[37]
15	P/T	Whole-gene deletion	ND	No	[42]
15	P/T	NM_000143.4(*FH*): c.1430_1437dup (p.Ser480Lysfs*6)	ND	No	[36]
17	P	c.994delA	ND	No	[28]
17	P	NM_000143.4(*FH*): c.1293del (p.Glu432fs)	ND	*MET*	[43]
17	NS	ND	ND	No	[44]

Cases of pediatric RCC in HLRCC/FH-TPS are reported from literature including age at diagnosis, RCC characteristics, and germline genetic testing results. Abbreviations: ND (not determined), NS (not specified, but patients with RCC were included with pathology outside typical FH-deficient-RCC-associated histopathology seen), P (papillary), T (tubulocystic), WES (whole-exome sequencing)

Given the rarity of pediatric RCC and emerging evidence that overall RCC is lower than historically reported, initiating renal MRI screening in childhood may not be justified. Current FH-TPS-HLRCC guidelines suggest annual renal MRI with contrast starting at age 10 years [4, 27, 45], with the goal of identifying all presymptomatic RCC. However, hereditary cancer guidelines typically balance early detection with considerations of cost, accessibility, and the burden of screening which have been questioned in pediatric patients with FH-TPS-HLRCC [17]. Previous cost-effectiveness analysis in FH-TPS-HLRCC using lifetime RCC risk of 21% demonstrated marginal utility of screening in pediatric patients (95% CI in pediatric age group had significant overlap with cost-ineffectiveness); thus, pediatric screening could easily be cost-ineffective if model assumptions for lifetime and pediatric risks were overestimates [46]. If pediatric RCC risk in FH-TPS-HLRCC is < 1%, then it could take ~1000 pediatric MRIs to detect a single case. In many other hereditary cancer conditions (and cancer screening in general), the age to initiate various screenings and interventions will not detect all cancers but is set to balance high detection with reasonable use of resources. As an example, colonoscopy for Lynch syndrome is recommended to start at age 20–25 years, but rare cases of colon cancer do occur in adolescents with Lynch syndrome prior to this age [47, 48].

Considering that FH-TPS-HLRCC is more common in the population than previously recognized, FH-deficient RCC risk is probably lower than early estimates, pediatric FH-deficient RCC is ultrarare, prior pediatric cases of RCC in FH-TPS-HLRCC are poorly evaluated for MINAS, children often require sedation for MRI, and MRI surveillance is costly, further analysis of pediatric screening is justified. A potential alternative would be to initiate annual MRI screening at 18 years old but apply a “10-year rule” that allows earlier screening in select families (initiating screening 10 years prior to the earliest age of RCC in a family if this results in a screening age less than 18 years), which is commonly used in several hereditary cancer syndromes to allow flexibility for earlier screening.

## Conclusions

Based on current understanding of phenotype-genotype correlation in *FH*, individuals heterozygous for LPV/PV in *FH* from a large health system were divided into groups (FH-ARC, FH-TPS-PHEO, and FH-TPS-HLRCC) and examined for relevant clinical features and RCC risks. Individuals with FH-TPS-HLRCC were diagnosed in about 1/3 of cases without a prior clinical suspicion or specific indication for FH-TPS testing. In patients with FH-TPS-HLRCC without a *FH*-specific test indication, many had subtle or less obvious features to suggest the diagnosis such as uterine leiomyomata. About 1/3 of patients with a *FH* test indication were undergoing testing for cascade reasons. Among patients with a clinical sign suggestive of FH-TPS-HLRCC, cutaneous leiomyomata were the most common clinical feature. Patients with FH-TPS-HLRCC are under-diagnosed, and inclusion of *FH* on a broad hereditary cancer gene panel appears to increase diagnosis. The cumulative incidence of RCC at age 70 years was 2.6% (95% CI 0.0-7.1%) in the FH-ARC group and 7.3% (95% CI 2.1–15.3%) in the FH-TPS-HLRCC group. All FH-deficient RCC occurred in the FH-TPS-HLRCC group, and the risk for RCC in this group was negligible under age 25 years. Universal MRI screening for RCC in adults with FH-TPS-HLRCC is appropriate, but the need for pediatric RCC screening in this condition requires further consideration as RCC risks are likely lower than previous estimates. Pediatric RCC screening in FH-TPS-HLRCC screening in select families with younger-onset RCC could be adopted using a “10-year rule” which is applied in many other hereditary cancer conditions to apply earlier screening in select families.

## Supplementary Information

Below is the link to the electronic supplementary material.

Supplementary Material 1

## Data Availability

The datasets used and/or analyzed during the current study are available from the corresponding author on reasonable request.

## Abbreviations

AR: Autosomal recessive
CNV: Copy number variant
CIF: Cumulative incidence function
FH: Fumarate hydratase
HLRCC: Hereditary leiomyomatosis and renal cell carcinoma
IHC: Immunohistochemistry
LPV: Likely pathogenic variant
MINAS: Multi-locus inherited neoplasia syndrome
PHEO: Pheochromocytoma
PV: Pathogenic variant
RCC: Renal cell carcinoma
2SC: s-2-succinocysteiene
TPS: Tumor predisposition syndrome
