# Supplementary material for: Renal cell carcinoma risk among individuals heterozygous for fumarate hydratase variants: further insights into genotype-phenotype correlations
Source: Hered Cancer Clin Pract. 2026 May 12;24:15. doi: 10.1186/s13053-026-00342-1 (PMC13335062; doi:10.1186/s13053-026-00342-1)
Supplement: Supplementary file 1 — Supplementary Material 1 [file 13053_2026_342_MOESM1_ESM.docx]

SUPPLEMENTAL FIGURE 1: *FH* Variant Assignment Process and Variant List


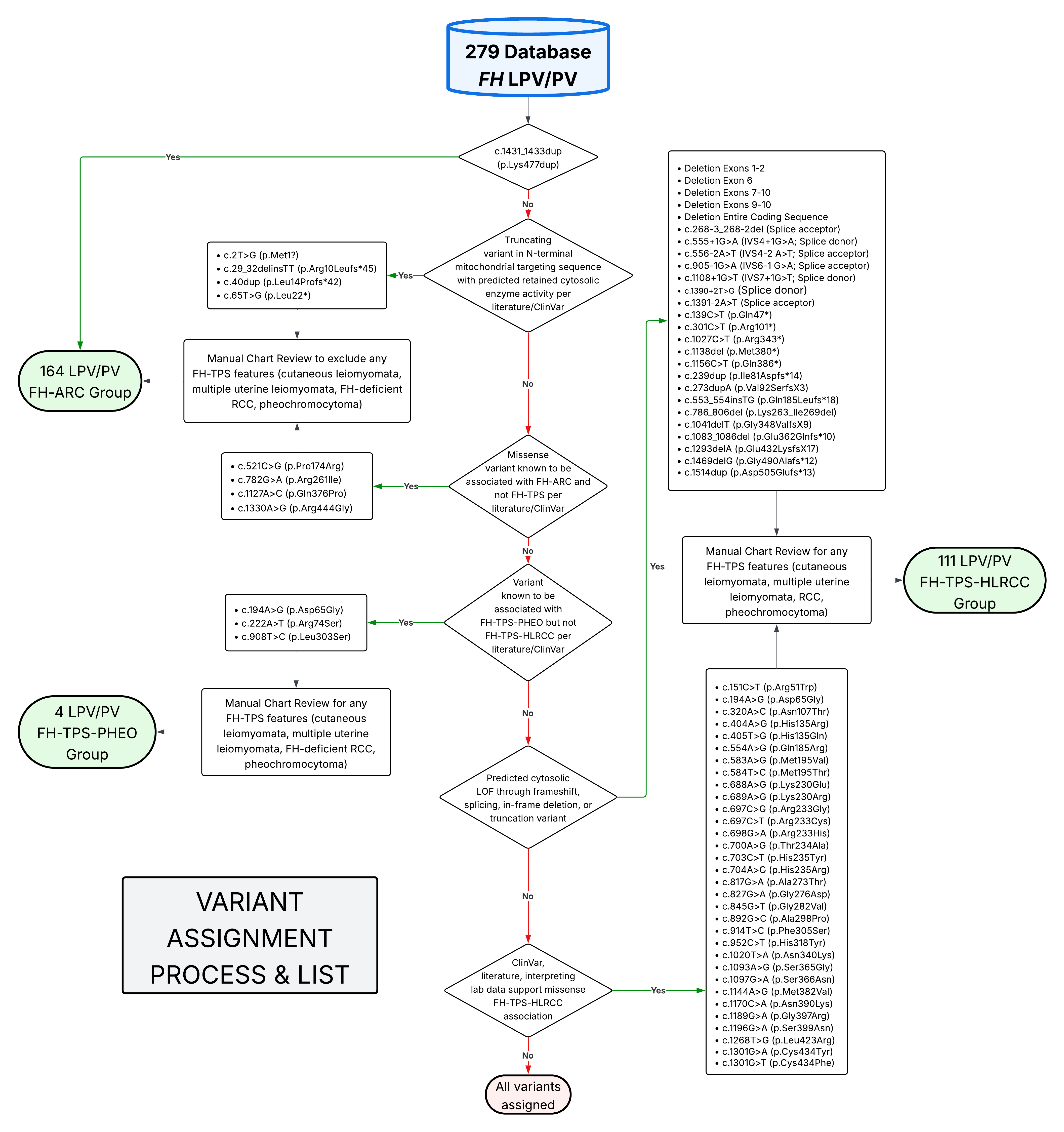


The process for patient/variant assignment into FH-ARC, FH-TPS-PHEO, and FH-TPS-HLRCC groups is shown along with specific variants assigned into each group.

SUPPLEMENTARY TABLE 1: LPV/PV *FH* Variants with ClinVar Identifiers

| **FH-ARC LPV/PV Variant List** | | | |
| --- | --- | --- | --- |
| Variant | ClinVar Identifier | ClinVar ID | Comment |
| c.1431_1433dup (p.Lys477dup) | NM_000143.4(*FH*):c.1431_1433dup (p.Lys477dup) | 42095 |  |
| c.2T>G (p.Met1?) | NM_000143.4(*FH*):c.2T>G (p.Met1Arg) | 429172 |  |
| c.29_32delinsTT (p.Arg10Leufs*45) | NM_000143.4(*FH*):c.29_ | 2675688 |  |
| c.40dup (p.Leu14Profs*42) | NM_000143.4(*FH*):c.40dup (p.Leu14fs) | 405920 |  |
| c.65T>G (p.Leu22*) | NM_000143.4(*FH*):c.65T>G (p.Leu22Ter) | 826506 |  |
|  |  |  |  |
| **FH-TPS-PHEO LPV/PV Variant List** | | | |
| Variant | ClinVar Identifier | ClinVar ID | Comment |
| c.194A>G (p.Asp65Gly) | NM_000143.4(*FH*):c.194A>G (p.Asp65Gly) | 214390 | ClinVar variant review suggests FH-TPS-PHEO |
| c.222A>T (p.Arg74Ser) | NM_000143.4(*FH*):c.222A>T (p.Arg74Ser) | 485565 | PMID: 20301430 |
| c.908T>C (p.Leu303Ser) | NM_000143.4(*FH*):c.908T>C (p.Leu303Ser) | 41584 | PMID: 20301430 |
|  |  |  |  |
| **FH-TPS-HLRCC LPV/PV Predicted LOF List** | | | |
| Variant | ClinVar Identifier | ClinVar ID | Comment |
| Deletion Exons 1-2 | None | N/A | Invitae pathogenic variant. Invitae variant description: This variant is a gross deletion of the genomic region encompassing exon(s) 1-2 of the *FH* gene, which includes the initiator codon. This deletion extends beyond the assayed region for this gene and therefore may encompass additional genes. It is expected to result in an absent or disrupted protein product. Loss-of-function variants in *FH* are known to be pathogenic (PMID: 11865300, 21398687). This variant has not been reported in the literature in individuals affected with FH-related conditions. For these reasons, this variant has been classified as Pathogenic. |
| Deletion Exon 6 | None | N/A | Invitae pathogenic variant. Invitae variant description: This variant is a gross deletion of the genomic region encompassing exon(s) 6 of the *FH* gene. This deletion is out-of-frame, and is expected to create a premature termination codon and result in an absent or disrupted protein product. Loss-of-function variants in *FH* are known to be pathogenic (PMID: 11865300, 21398687). A similar copy number variant has been observed in individual(s) with leiomyomatosis and renal cell cancer (PMID: 22382802). For these reasons, this variant has been classified as Pathogenic. |
| Deletion Exons 7-10 | None | N/A | Invitae pathogenic variant. Invitae variant description: A similar copy number variant has been observed in individual(s) with hereditary leiomyomatosis and renal cell carcinoma (PMID: 28196407). This variant disrupts a region of the FH protein in which other variant(s) (p.Gly490Alafs*12, p.Leu492Hisfs*6, p.Glu495Valfs*2, and p.Trp500*) have been determined to be pathogenic (PMID: 9635293, 12772087, 16597677, 21398687, 21404119; Invitae). This suggests that this is a clinically significant region of the protein, and that variants that disrupt it are likely to be disease-causing. |
| Deletion Exons 9-10 | None | N/A | Invitae pathogenic variant. Invitae variant description: Although no functional studies have assayed the effect of this particular deletion on FH protein function or stability, several truncating variants in the last exon (p.Gly490Alafs12, p.Leu492Hisfs6, p.Glu495Valfs2, and p.Trp500) have been determined to be likely pathogenic (PMID: 12772087, 21404119, 16597677, 9635293, 21398687, Invitae database). This suggests that the C-terminal region of the FH protein is critical for the proper protein function. |
| Deletion Entire Coding Sequence) | None | N/A | Invitae pathogenic variant. Invitae variant description: A gross deletion of the genomic region encompassing the full coding sequence of the *FH* gene has been identified. Loss-of-function variants in *FH* are known to be pathogenic (PMID: 11865300, 21398687). The boundaries of this event are unknown as they extend beyond the assayed region for this gene and therefore may encompass additional genes. A similar copy number variant has been observed in individual(s) with hereditary leiomyomatosis and renal cell carcinoma (HLRCC) syndrome and fumarate deficiency (PMID: 11865300, 12761039, 21398687, 22069215, 28300276). |
| c.268-3_268-2del (Splice acceptor) | NM_000143.4(*FH*):c.268-3_268- | 1499090 |  |
| c.555+1G>A (IVS4+1G>A; Splice donor) | NM_000143.4(*FH*):c.555+1G>A | 449388 |  |
| c.556-2A>T (IVS4-2 A>T; Splice acceptor) | NM_000143.4(*FH*):c.556-2A>T | 393566 |  |
| c.905-1G>A (IVS6-1 G>A; Splice acceptor) | NM_000143.4(*FH*):c.905-1G>A | 208374 |  |
| c.1108+1G>T (IVS7+1G>T; Splice donor) | NM_000143.4(*FH*):c.1108+1G>T | 372365 |  |
| c.1390+2T>G (Splice donor) | NM_000143.4(*FH*):c.1390+2T>G | 4725426 |  |
| c.1391-2A>T (Splice acceptor) | NM_000143.4(*FH*):c.1391-2A>T | 214423 |  |
| c.139C>T (p.Gln47*) | NM_000143.4(*FH*):c.139C>T (p.Gln47Ter) | 214389 |  |
| c.301C>T (p.Arg101*) | NM_000143.4(*FH*):c.301C>T (p.Arg101Ter) | 16232 |  |
| c.1027C>T (p.Arg343*) | NM_000143.4(*FH*):c.1027C>T (p.Arg343Ter) | 16235 |  |
| c.1138del (p.Met380*) | NM_000143.4(*FH*):c.1138del (p.Ala379_Met380insTer) | 572058 |  |
| c.1156C>T (p.Gln386*) | NM_000143.4(*FH*):c.1156C>T (p.Gln386Ter) | 2767087 |  |
| c.239dup (p.Ile81Aspfs*14) | NM_000143.4(*FH*):c.239dup (p.Ile81fs) | 393558 |  |
| c.273dupA (p.Val92SerfsX3) | NM_000143.4(*FH*):c.273dup (p.Val92fs) | 1795415 |  |
| c.553_554insTG (p.Gln185Leufs*18) | NM_000143.4(*FH*):c.553_554insTG (p.Gln185fs) | 393565 |  |
| c.786_806del (p.Lys263_Ile269del) | NM_000143.4(*FH*):c.786_806del (p.Lys263_Ile269del) | 185496 |  |
| c.1041delT (p.Gly348ValfsX9) | NM_000143.4(*FH*):c.1041del (p.Gly348fs) | 393576 |  |
| c.1083_1086del (p.Glu362Glnfs*10) | NM_000143.4(*FH*):c.1083_1086del (p.Glu362fs) | 214399 |  |
| c.1293delA (p.Glu432LysfsX17) | NM_000143.4(*FH*):c.1293del (p.Glu432fs) | 92452 |  |
| c.1469delG (p.Gly490Alafs*12) | NM_000143.4(*FH*):c.1469del (p.Gly490fs) | 393582 |  |
| c.1514dup (p.Asp505Glufs*13) | NM_000143.4(*FH*):c.1514dup (p.Asp505fs) | 3652241 |  |
|  |  |  |  |
| **FH-TPS-HLRCC LPV/PV Missense Variant List** | | | |
| Variant | ClinVar Identifier | ClinVar ID | Comment |
| c.151C>T (p.Arg51Trp) | NM_000143.4(*FH*):c.151C>T (p.Arg51Trp) | 649446 |  |
| c.194A>G (p.Asp65Gly) | NM_000143.4(*FH*):c.194A>G (p.Asp65Gly) | 214390 |  |
| c.320A>C (p.Asn107Thr) | NM_000143.4(*FH*):c.320A>C (p.Asn107Thr) | 92455 |  |
| c.404A>G (p.His135Arg) | NM_000143.4(*FH*):c.404A>G (p.His135Arg) | 186284 |  |
| c.405T>G (p.His135Gln) | NM_000143.4(*FH*):c.405T>G (p.His135Gln) | 940083 |  |
| c.554A>G (p.Gln185Arg) | NM_000143.4(*FH*):c.554A>G (p.Gln185Arg) | 214433 |  |
| c.583A>G (p.Met195Val) | NM_000143.4(*FH*):c.583A>G (p.Met195Val) | 460368 |  |
| c.584T>C (p.Met195Thr) | NM_000143.4(*FH*):c.584T>C (p.Met195Thr) | 214373 |  |
| c.688A>G (p.Lys230Glu) | NM_000143.4(*FH*):c.688A>G (p.Lys230Glu) | 214375 |  |
| c.689A>G (p.Lys230Arg) | NM_000143.4(*FH*):c.689A>G (p.Lys230Arg) | 429176 |  |
| c.697C>G (p.Arg233Gly) | NM_000143.4(*FH*):c.697C>G (p.Arg233Gly) | 826742 |  |
| c.697C>T (p.Arg233Cys) | NM_000143.4(*FH*):c.697C>T (p.Arg233Cys) | 141355 |  |
| c.698G>A (p.Arg233His) | NM_000143.4(*FH*):c.698G>A (p.Arg233His) | 16236 |  |
| c.700A>G (p.Thr234Ala) | NM_000143.4(*FH*):c.700A>G (p.Thr234Ala) | 184555 |  |
| c.703C>T (p.His235Tyr) | NM_000143.4(*FH*):c.703C>T (p.His235Tyr) | 214376 |  |
| c.704A>G (p.His235Arg) | NM_000143.4(*FH*):c.704A>G (p.His235Arg) | 844336 |  |
| c.817G>A (p.Ala273Thr) | NM_000143.4(*FH*):c.817G>A (p.Ala273Thr) | 214377 |  |
| c.827G>A (p.Gly276Asp) | NM_000143.4(*FH*):c.827G>A (p.Gly276Asp) | 1067347 |  |
| c.845G>T (p.Gly282Val) | NM_000143.4(*FH*):c.845G>T (p.Gly282Val) | 960404 |  |
| c.892G>C (p.Ala298Pro) | NM_000143.4(*FH*):c.892G>C (p.Ala298Pro) | 198045 |  |
| c.914T>C (p.Phe305Ser) | NM_000143.4(*FH*):c.914T>C (p.Phe305Ser) | 823050 |  |
| c.952C>T (p.His318Tyr) | NM_000143.4(*FH*):c.952C>T (p.His318Tyr) | 92458 |  |
| c.1020T>A (p.Asn340Lys) | NM_000143.4(*FH*):c.1020T>A (p.Asn340Lys) | 92447 |  |
| c.1093A>G (p.Ser365Gly) | NM_000143.4(*FH*):c.1093A>G (p.Ser365Gly) | 214374 |  |
| c.1097G>A (p.Ser366Asn) | NM_000143.4(*FH*):c.1097G>A (p.Ser366Asn) | 214419 |  |
| c.1144A>G (p.Met382Val) | NM_000143.4(*FH*):c.1144A>G (p.Met382Val) | 265148 |  |
| c.1170C>A (p.Asn390Lys) | NM_000143.4(*FH*):c.1170C>A (p.Asn390Lys) | 1303417 |  |
| c.1189G>A (p.Gly397Arg) | NM_000143.4(*FH*):c.1189G>A (p.Gly397Arg) | 214422 |  |
| c.1196G>A (p.Ser399Asn) | NM_000143.4(*FH*):c.1196G>A (p.Ser399Asn) | 860747 |  |
| c.1268T>G (p.Leu423Arg) | NM_000143.4(*FH*):c.1268T>G (p.Leu423Arg) | 214424 |  |
| c.1301G>A (p.Cys434Tyr) | NM_000143.4(*FH*):c.1301G>A (p.Cys434Tyr) | 92453 |  |
| c.1301G>T (p.Cys434Phe) | NM_000143.4(*FH*):c.1301G>T (p.Cys434Phe) | 3719052 |  |

SUPPLEMENTARY METHODS: Coding Strategy to Identify RCC Cases in Epic-based EMR

Patient encounter diagnoses were queried for patients with LPV/PV *FH* variants in the Kaiser Permanente Epic-based electronic medical record via Epic Clarity. The earliest encounter (contact date) with an RCC diagnosis was used as the date on which RCC was confirmed. Manual medical chart review confirmed all RCC cases in this study for accuracy. If two or more RCC diagnoses were associated with the same patient on the same contact date, the diagnosis listed as “primary” was chosen. None of the patients in this study had more than one RCC diagnosis on the same contact date.

The following are a list of all diagnoses identified as “events” in this study:

| Description | ICD10 Code |
| --- | --- |
| RENAL CELL CARCINOMA, RIGHT KIDNEY | C64.1 |
| KIDNEY CANCER, LEFT KIDNEY | C64.2 |
| LOCALIZED KIDNEY CANCER, UNSPECIFIED SITE | C64.9 |
| KIDNEY CANCER, LEFT KIDNEY | C64.2 |
| RENAL CELL CARCINOMA, RIGHT KIDNEY | C64.1 |
| KIDNEY CANCER, LEFT KIDNEY | C64.2 |
| RENAL CELL CARCINOMA, RIGHT KIDNEY | C64.1 |
| TRANSITIONAL CELL CARCINOMA, LEFT RENAL PELVIS | C65.2 |
| RENAL CELL CARCINOMA, LEFT KIDNEY | C64.2 |

In total, 81 diagnoses related to renal cancer were queried including ICD10 codes related to active or history of renal cancer diagnoses.

The query below was used to identify all RCC cases:

| select s.pat_id, s.pat_mrn_id, s.first_renal_cancer, s.cnt_num_renal_ca_listed, count(ptdx.pat_id) as cnt,  count(case when ptdx.primary_dx_yn = 'Y' then ptdx.pat_id end) as y_cnt -- it is possible two renal cancer diagnoses are associated to the same encounter. will use the primary diagnosis if there are 2 or more.  from      (      select fh.pat_id, fh.pat_mrn_id, min(ptdx.contact_date) as first_renal_cancer, count(ptdx.DX_ID) as cnt_num_renal_ca_listed  --acquire earliest date there was a renal cancer diagnosis      from FH_CDNA_CARRIERS fh --Table of all patients with known *FH* (derived elsewhere)      left join HCCLSC.PAT_ENC_DX ptdx      on fh.pat_id = ptdx.pat_id      inner JOIN RENAL_CANCER_DX_ID  ON ptdx.DX_ID = RENAL_CANCER_DX_ID.DX_ID      group by fh.pat_id, fh.pat_mrn_id      ) s    left join HCCLSC.PAT_ENC_DX ptdx  on s.pat_id = ptdx.pat_id  and s.first_renal_cancer = ptdx.contact_date -- acquire all diagnoses associated with the first onset of a renal cancer.  inner JOIN RENAL_CANCER_DX_ID  ON ptdx.DX_ID = RENAL_CANCER_DX_ID.DX_ID  group by s.pat_id, s.pat_mrn_id, s.first_renal_cancer, s.cnt_num_renal_ca_listed |
| --- |
